# Aberrant DNA Methylation of *rDNA* and *PRIMA1* in Borderline Personality Disorder

**DOI:** 10.3390/ijms17010067

**Published:** 2016-01-05

**Authors:** Stefanie Teschler, Julia Gotthardt, Gerhard Dammann, Reinhard H. Dammann

**Affiliations:** 1 Institute for Genetics, Justus-Liebig-University Giessen, D-35392 Giessen, Germany; stefanie.teschler@gen.bio.uni-giessen.de (S.T.); Julia.Gotthardt@bio.uni-giessen.de (J.G.); 2 Psychiatric Hospital, Psychiatric Services of Thurgovia, CH-8596 Münsterlingen, Switzerland and Department of Psychiatry, Paracelsus Medical University, A-5020 Salzburg, Austria; gerhard.dammann@stgag.ch

**Keywords:** borderline personality disorder, ribosomal RNA gene, proline rich membrane anchor gene, epigenetics, DNA methylation

## Abstract

Borderline personality disorder (BPD) is a serious psychic disease with a high risk for suicide. DNA methylation is a hallmark for aberrant epigenetic regulation and could be involved in the etiology of BPD. Previously, it has been reported that increased DNA methylation of neuropsychiatric genes is found in the blood of patients with BPD compared to healthy controls. Here, we analyzed DNA methylation patterns of the *ribosomal RNA* gene (*rDNA* promoter region and 5′-external transcribed spacer/5′ETS) and the promoter of the *proline rich membrane anchor 1* gene (*PRIMA1*) in peripheral blood samples of 24 female patients (mean age (33 ± 11) years) diagnosed with DSM-IV BPD and in 11 female controls (mean age (32 ± 7) years). A significant aberrant methylation of *rDNA* and *PRIMA1* was revealed for BPD patients using pyrosequencing. For the promoter *of PRIMA1*, the average methylation of six CpG sites was 1.6-fold higher in BPD patients compared to controls. In contrast, the methylation levels of the *rDNA* promoter region and the 5′ETS were significantly lower (0.9-fold) in patients with BPD compared to controls. Thus, for nine CpGs located in the *rDNA* promoter region and for four CpGs at the 5′ETS decreased methylation was found in peripheral blood of patients compared to controls. Our results suggest that aberrant methylation of *rDNA* and *PRIMA1* is associated with the pathogenesis of BPD.

## 1. Introduction

Typical features of borderline personality disorder (BPD) are impulsive aggression, emotional dysregulation, repeated self-injury, but also chronic suicidality, and, therefore, BPD patients are often under clinical treatments [1,2]. Different signs of psychosocial impairment have been reported and the 50-times increased suicidality causes a 10% mortality rate in BPD patients [3] Treatments like semi-structural interviews are of benefit for BPD patients [4]. BPD seems to be less stable over time than expected for personality disorders [5]. For the etiology of BPD, little is known; however, epigenetic and genetic factors [6,7,8] and physical and sexual abuse during childhood [9] have been associated with the pathogenesis of BPD.

Various forms of epigenetic regulation at the levels of DNA methylation, histone modification, and non-coding RNAs (ncRNAs) can modulate transcriptional and translational events required for memory processes [10]. About 60% of genes harbor CpG- and GC-rich DNA sequences in their 5′ regulatory region, which are termed CpG island promoters [11]. Active genes are associated with an open and unmethylated CpG island promoter and hypermethylated CpG islands are correlated with epigenetically silenced genes [12,13]. By changing the cellular profile in the brain's emotional, reward, and memory circuits, these epigenetic modifications have also been linked to perseverant, pathogenic memories typical for post-traumatic stress disorder (PTSD), major depressive disorder (MDD) and BPD [10,14]. Since environmental stimuli can influence epigenetic modifications, like DNA methylation, these aberrant marks could be associated with the development of anxiety [15], suicidality [16], and vulnerability for stress [17]. Previously, significant aberrant DNA methylation of *serotonin receptor 2A (HTR2A)*, *monoamine oxidase A and B (MAOA and MAOB)*, *glucocorticoid receptor (NR3C1)*, *brain derived neurotrophic factor (BDNF)*, *amyloid beta precursor protein-binding family A member 2 and 3 (APBA2 and APBA3)*, *KCNQ1*, *MCF2*, and *ninjurin 2 (NINJ2)* has been reported [18,19,20,21,22,23,24,25,26,27,28,29]. Changes in DNA methylation range from 1.1- to 1.5-fold increased methylation in the blood of patients with BPD. Especially, *NR3C1* methylation was positively associated with childhood maltreatment and clinical severity in BPD [22,23].

Recently, a genome-wide screen for DNA methylation has been performed for the major depressive disorder (MDD) [14]. Sabunciyan *et al.* compared the methylation pattern of 39 postmortem frontal cortex MDD samples to 26 controls. They reported increased methylation (12%–15%) of the *proline rich membrane anchor 1* gene (*PRIMA1*) and a concomitant decrease in gene expression [14]. *PRIMA1* functions by organizing acetylcholinesterase into tetramers, and by anchoring these tetramers at neural cell membranes [30,31]. Increased methylation of *PRIMA1* results in decreased enzyme function and increased cholinergic transmission, consistent with a role of the cholinergic circuit in different psychiatric disorders [14,32]. In BPD, the activation of the cholinergic system in peripheral blood cells of patients has been reported [33].

Epigenetic deregulation of *rRNA* genes by aberrant DNA methylation has been found in different human diseases, including cancer [13,34,35]. In psychiatric diseases, increased methylation of the regulatory CpG island promoter of *rRNA* genes has been reported in the brain of suicide persons and this has been correlated with reduced *rRNA* levels [36].

In our study, we investigated the methylation levels of the promoter region and 5′ETS of the ribosomal RNA genes and of the promoter of *PRIMA1* in peripheral blood from female patients diagnosed with BPD and age matched control samples from female students without prior history of mental disorders. Since a promoter harbors the main regulatory sequences of the associated gene, we analyzed the level of DNA methylation of this region via bisulfite pyrosequencing. Here, we report that a significant aberrant promoter methylation of *rDNA* and *PRIMA1* was observed in the blood of BPD patients compared to controls.

## 2. Results and Discussion

### 2.1. Hypomethylation of rDNA in Borderline Personality Disorder

**Figure 1:**
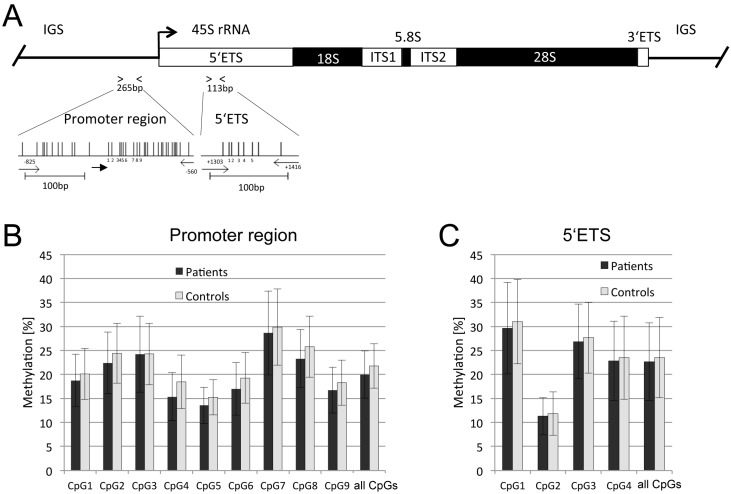
Methylation analysis of rDNA in blood from borderline personality disorder (BPD) patients and control persons. (**A**) Map of a human *rRNA* gene. The transcription start site of the 45S *rRNA* is marked with an arrow; The *rRNA* gene is organized in 5′ external transcribed spacer (5′ETS), *18S rRNA*, internal transcribed spacer 1 (*ITS1*), *5.8S rRNA*, *ITS2*, *28S rRNA*, *3ʹETS* and intergenic spacer (IGS). PCR products for bisulfite sequencing are depicted. Individual CpGs are indicated by vertical lines and the analyzed CpGs for each region are numbered. Primers are marked with arrows. Graphics were generated with the Python *vs.* Cobra program (https://launchpad.net/python.vs.cobra) and scale bars indicate 100 bp. Methylation levels of nine CpGs located in the distal promoter (**B**); and four CpGs in the 5′ETS (**C**) were obtained by bisulfite pyrosequencing. CpGs indicate the mean methylation of the analyzed region.

Previously, an increased methylation throughout the *rDNA* CpG island promoter has been revealed in the brain of suicide persons [36]. Especially, borderline personality disorder (BPD) is associated with a 50-times increase in suicidal rate compared to the general population. Since we have already reported aberrant methylation of several genes in the blood of BPD patients [19,28], we aimed to reveal the methylation status of *rDNA* in peripheral blood of BPD patients. Human *rDNA* consists of hundreds of *rDNA* repeats of CpG-rich *rRNA* genes (Figure 1A). We analyzed the methylation of nine CpGs located in the distal promoter region and four CpGs in the 5′ external transcribed spacer (5′ETS) (Figure 1B,C). Data on methylation levels were obtained from blood samples of 11 female controls and 24 female patients with BPD (Table 1).

**Figure 2:**
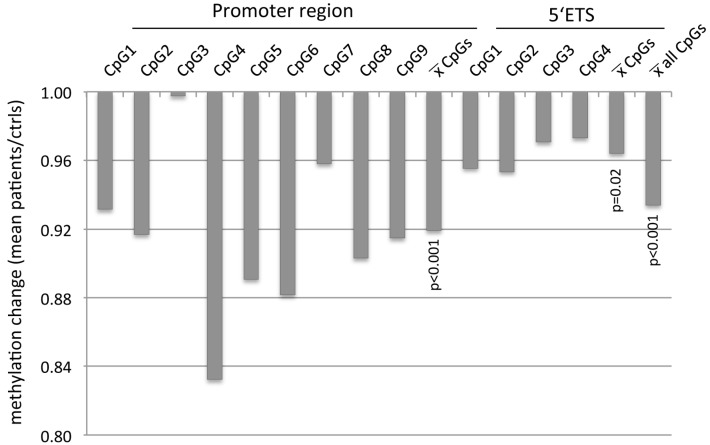
Hypomethylation of *rDNA* in borderline personality disorder patients. The quotient between mean methylation levels at individual CpG sites and combined sites for the promoter region and 5′ETS in BPD patients and controls (ctrls) were calculated and plotted. Statistical analysis was performed with the two-tailed, paired *t*-test comparing the mean methylation of matching CpG sites in a specified region.

For the rDNA promoter region, methylation levels in patients ranged from 10% to 30% with following percentages for CpG1: 18.7%, CpG2: 22.4%, CpG3: 24.2%, CpG4: 15.3%, CpG5: 13.5%, CpG6: 17.0%, CpG7: 28.6%, CpG8: 23.3% and 16.7% for CpG9 (Figure 2B). In controls slightly higher methylation levels were observed for the analyzed CpGs: 20.1% at CpG1; CpG2: 24.4%; CpG3: 24.3%; CpG4: 18.4%; CpG5: 15.2%; CpG6: 19.3%; CpG7: 29.9%; CpG8: 25.8% and CpG9: 18.3% (Figure 2B).

**Table 1:** Summarized data of the analyzed patients with BPD and control persons ^a^.

Category	BPD Patients (*n* = 24)	Control Persons (*n* = 11)
Female	24	11
Mean age (±SD)	33 ± 11	32 ± 7
DSM-IV criteria 1 ^b^	75% (18/24)	0% (*p* ≤ 0.001) ^c^
DSM-IV criteria 2 ^b^	54% (13/24)	0% (*p* ≤ 0.003) ^c^
DSM-IV criteria 3 ^b^	92% (22/24)	0% (*p* ≤ 0.001) ^c^
DSM-IV criteria 4 ^b^	63% (15/24)	0% (*p* ≤ 0.001) ^c^
DSM-IV criteria 5 ^b^	88% (21/24)	0% (*p* ≤ 0.001) ^c^
DSM-IV criteria 6 ^b^	88% (21/24)	0% (*p* ≤ 0.001) ^c^
DSM-IV criteria 7 ^b^	58% (14/24)	0% (*p* ≤ 0.002) ^c^
DSM-IV criteria 8 ^b^	63% (15/24)	0% (*p* ≤ 0.001) ^c^
DSM-IV criteria 9 ^b^	54% (13/24)	0% (*p* ≤ 0.003) ^c^
Observed positive diagnosis	100% (24/24)	0% (*p* ≤ 0.001) ^c^
Acute self injuring behavior (ASIB) ^a^	63% (15/24)	0% (*p* ≤ 0.002) ^c^
Prior self injuring behavior (PSIB) ^a^	88% (21/24)	0% (*p* ≤ 0.001) ^c^
Suicide background (SB) ^a^	83% (20/24)	0% (*p* ≤ 0.001) ^c^
Nicotine consumption (NC) ^a^	71% (17/24)	36% (4/11) (n.s.) ^c^
Alcohol abuse (AA) ^a^	25% (6/24)	0% (n.s.) ^c^
Additional drug abuse (ADA) ^a^	17% (4/24)	0% (n.s.) ^c^
Prior traumatic experience (PTE) ^a^	63% (15/24)	0% (*p* ≤ 0.001) ^c^

^a^ More details are listed in Table 2 and [19]; ^b^ DSM-IV: A pervasive pattern of instability of interpersonal relationships, self-image, and affects, and marked impulsivity beginning by early adulthood and present in a variety of contexts, as indicated by five (or more) of the following: criteria (crit.) 1: frantic efforts to avoid real or imagined abandonment. Note 1: Do not include suicidal or self-mutilating behavior covered in crit. 5; crit. 2: a pattern of unstable and intense interpersonal relationships characterized by alternating between extremes of idealization and devaluation; crit. 3: identity disturbance: markedly and persistently unstable self image or sense of self; crit. 4: impulsivity in at least two areas that are potentially self-damaging (e.g., spending, sex, substance abuse, reckless driving, binge eating); Note 2: Do not include suicidal or self-mutilating behavior covered in crit. 5; crit. 5: recurrent suicidal behavior, gestures, or threats, or self-mutilating behavior; crit. 6: affective instability due to a marked reactivity of mood (e.g., intense episodic dysphoria, irritability, or anxiety usually lasting a few hours and only rarely more than a few days); crit. 7: chronic feelings of emptiness; crit. 8: inappropriate, intense anger or difficulty controlling anger (e.g., frequent displays of temper, constant anger, recurrent physical fights; crit. 9: transient, stress-related paranoid ideation or severe dissociative symptoms; ^c^ two tailed Fisher exact probability test: n.s. not significant (*p* > 0.05).

Similarly, *5′ETS* methylation levels of four CpG sites ranged from 10% to 30% in patients and controls (Figure 1C). Also here, in BPD patients a slightly lower level of methylation was found for all analyzed CpGs compared to controls (CpG1: 29.6% and 31.0%, CpG2: 11.3% and 11.8%, CpG3: 26.9% and 27.7%, CpG4: 22.9% and 23.5% (Figure 1C)). Interestingly, the mean methylation level was lower for all analyzed CpGs in the distal promoter region and 5′ETS in BPD patients (20% and 23%) compared to control patients (22% and 24%, respectively; Figure 1). However, due to inter- and intra-individual variations in *rDNA* methylation, a large variability was observed in peripheral blood. Utilizing a paired *t*-test analysis of the mean methylation of nine CpGs in the distal promoter, a significant decrease was detected in BPD samples compared to controls (Figure 2). For the promoter, a 0.92-fold lower methylation in BPD patients compared to controls was revealed (*p* < 0.001). In the 5′ETS, a similar trend (*p* = 0.02) with a 0.96-fold lower methylation level in BPD patients was observed (Figure 2). Thus, for both regions together, an average 0.93-fold lower methylation was found (*p* < 0.001; Figure 2).

Previously, aberrant DNA methylation of *rRNA* genes has been reported for different human diseases, including dementia [34,35]. For psychiatric diseases, *rDNA* was significantly higher methylated throughout the promoter and 5′ regulatory region in the brain of suicide subjects, consistent with reduced *rRNA* expression in the hippocampus [36]. In our study, however, we observed a hypomethylation of the distal *rDNA* promoter and 5′ETS region. This difference could be attributed to the analyzed regions or to distinct tissues analyzed. For example, in hepatocellular carcinoma, hypomethylation of the *rDNA* promoter has been revealed [37]. In other cancer tissues (e.g., breast, lung, colorectal and endometrial) hypermethylation of *rRNA* genes compared to normal tissues has been observed [13,34,38,39,40]. Thus, it will also be interesting to analyze the brain-specific methylation of *rDNA* in BPD patients. Bacalini *et al.* reported a considerable inter-individual variation in methylation levels [38], which was also seen in the blood of BPD patients and control samples. Therefore, a considerable standard deviation was observed. In eukaryotes, only a subset of *rDNA* is actively transcribed and the other fraction of repeats is epigenetically silenced [41,42,43]. We and others have shown that the inactive copies are packed into nucleosomes and contain methylated promoter sequences [44,45,46], whereas the transcribed *rRNA* genes are free of nucleosomes and the *rRNA* promoter is unmethylated [46,47,48]. Therefore, our data as well as other data suggest that, on average, 70% to 90% of the human *rDNA* repeats are epigenetically inactive [13,38].

### 2.2. Hypermethylation of PRIMA1 in Borderline Personality Disorder

**Figure 3:**
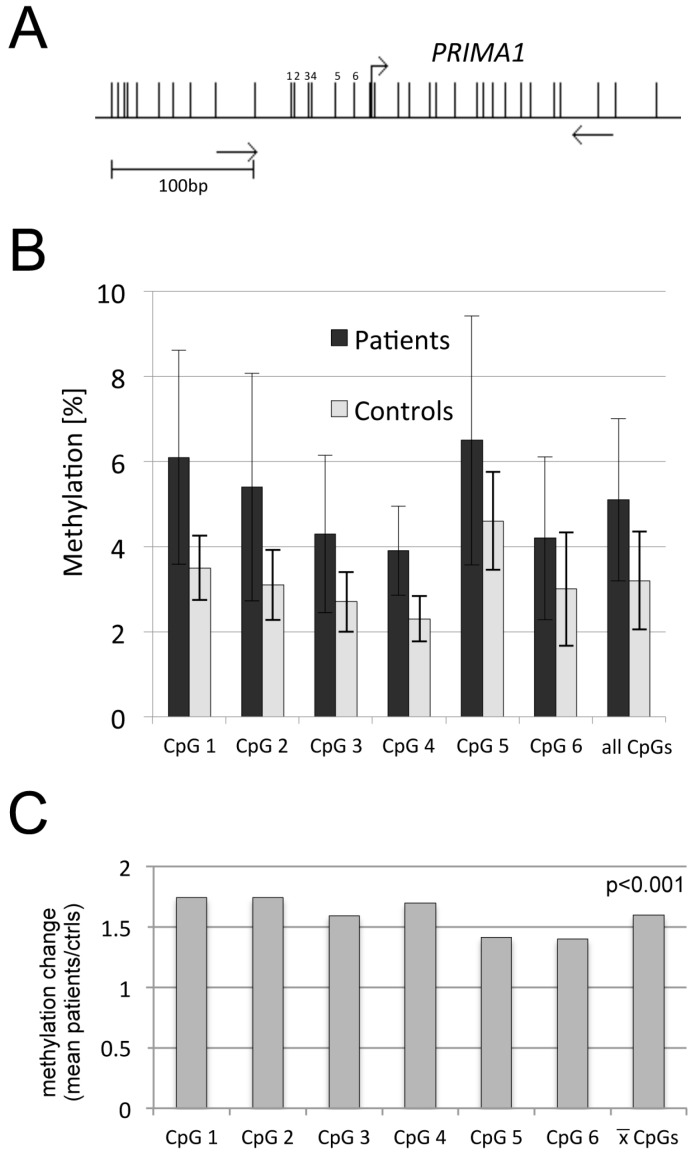
Methylation analysis of *PRIMA1* in blood samples of borderline personality disorder patients and control persons (**A**) Map of the *PRIMA1* gene. Individual CpGs are indicated by vertical lines and the six analyzed CpGs are numbered. Primers are marked with arrows. For details also see Figure 1; (**B**) Methylation levels of six CpGs located in the proximal promoter region were obtained by bisulfite pyrosequencing. All CpGs indicate the mean methylation of the analyzed region; (**C**) The quotients between mean methylation levels at individual CpG sites and the sites combined for *PRIMA1* in patients and controls (ctrls) were calculated and plotted. Statistical analysis was performed with the two-tailed, paired *t*-test comparing the mean methylation of matching CpG sites in a specific region.

In a genome-wide DNA methylation scan, increased methylation and a decreased gene expression of the *proline rich membrane anchor 1* gene (*PRIMA1*) have been reported in the major depressive disorder (MDD) [14]. Thus, we aimed to reveal the methylation status of *PRIMA1* in BPD. The promoter of *PRIMA1* consists of one CpG island.We analyzed six CpGs upstream of the transcription initiation site by bisulfite pyrosequencing (Figure 3A). For BPD patients, methylation levels were as following: for CpG1 6.1%, CpG2: 5.4%, CpG3: 4.3%, CpG4: 3.8%, CpG5: 6.5% and 4.2% for CpG6 (Figure 3B). Due to inter-individual variations a large variability was observed. In controls lower methylation levels of *PRIMA1* at all analyzed CpGs were observed (3.5% at CpG1; CpG2: 3.1%; CpG3: 2.7%; CpG4: 4.6%; CpG5 and CpG6: 3.0% (Figure 3B)). Thus, the mean methylation is 1.6-fold higher in BPD patients (5.1%) compared to controls (3.2%) (Figure 3B,C). Since the increased methylation was present at all six CpG sites (1.4- to 1.7-fold increase), this trend was significant in a statistical analysis with a paired *t*-test by dissecting the mean methylation of the six CpGs (*p* < 0.001; Figure 3C). The low methylation level of the *PRIMA1* promoter is consistent with its localization in a CpG island region which in turn are rather unmethylated in tissues [11]. We and others have revealed low methylation levels (<10% methylation) at the CpG islands of the *serotonin receptor 2A (HTR2A)*, *glucocorticoid receptor (NR3C1)* and *brain derived neurotrophic factor (BDNF)* [19,26,29].

In the major depressive disorder (MDD), an increased methylation of *PRIMA1* was previously reported [14]. Sabunciyan *et al.* compared the methylation pattern of postmortem frontal cortex MDD samples to controls. Hereby, they revealed an increased methylation (1.2-fold) at six CpGs within the coding region of *PRIMA1* [14]. Here, we detected an increased methylation (1.6-fold) in the proximal promoter region (Figure 3). Since only two patients (8%) had comorbid major depressive disorder (Table 2: F33.1), but all patients (100%) were positively diagnosed for BPD (Table 1), the observed difference in the methylation level cannot simply be attributed to comorbidity. Transcriptional regulation of *PRIMA1* has been linked to the MAP kinase pathway [49]. The gene product of PRIMA1 functions by organizing acetylcholinesterase (AChE) into tetramers, and by anchoring AChE at membranes of neural cells [50,51,52]. The membrane-bound form of AChE is the major fraction in the mammalian brain [30]. AChE is a serine protease, which hydrolyses the neurotransmitter acetylcholine into acetate and choline, thereby terminating neurotransmission [53]. PRIMA1 is of substantial biological interest in psychiatric disorders because of its relationship to neurotransmission. Interestingly, when BPD patients were treated with physostigmine in order to analyze AChE system responsiveness, the depressive response to physostigmine was enhanced in BPD patients [54]. Physostigmine is a reversible inhibitor of AChE [53]. Thus, it will be interesting to analyze the levels of membrane bound AChE and PRIMA1 in the brains of BPD patients.

## 3. Experimental Section

### 3.1. Tissue Samples

Whole blood samples of 24 female BPD patients and 11 age matched female controls were obtained from the Psychiatric Hospital in Münsterlingen, Switzerland (Table 1 and Table 2) and are the same samples as previously described and utilized [19,28]. Diagnosis of BPD was established by an experienced psychiatrist (Gerhard Dammann) following SCID-II-Interview [55] for DSM-IV [56] diagnosis of personality disorders. All patients were diagnosed positively for BPD due to impulsivity, suicidality and depression found in patients (Table 1).

All patients were taking medications (mainly antidepressants and atypical antipsychotics). Due to ethical reasons it was not possible to stop medication. The normal control groups consisted of students without prior history of mental disorders [19,28]. Informed consent was obtained from all subjects. All experiments were performed in accordance with relevant guidelines and regulations. This study and all experiments were approved of by the local ethic committees: ethic committee of the Kanton Thurgau/CH, ethic committee of the University Halle/Germany and ethic committee of the Justus-Liebig University Giessen/Germany.

**Table 2:** Clincopathological parameters of borderline personality disorder (BPD) patients.

Patient No.	Age	Crit. 1	Crit. 2	Crit. 3	Crit. 4	Crit. 5	Crit. 6	Crit. 7	Crit. 8	Crit. 9	ASIB ^a^	PSIB ^a^	SB ^a^	NC ^a^	AA ^a^	ADA ^a^	PTE ^a^	Co-Diagnosis ^a^
1	40	+	−	+	+	+	+	+	−	−	+	+	−	−	−	−	+	–
2	33	+	−	+	+	+	+	+	+	+	+	+	+	+	−	−	−	–
3	24	+	+	+	+	+	+	+	+	+	−	+	+	+	−	−	+	–
4	40	+	−	+	+	−	+	+	+	+	−	+	+	+	−	−	−	F10
5	52	+	−	+	+	+	−	+	−	+	−	−	+	−	−	−	+	–
6	17	+	+	+	+	+	+	+	+	+	+	+	+	+	−	−	+	–
7	28	+	−	+	+	+	+	−	+	+	+	+	+	−	−	−	+	F42.2
8	47	+	−	+	−	+	+	+	−	−	−	+	+	−	−	−	−	Depression; anorexia
9	51	+	−	+	−	+	+	+	−	−	−	+	+	+		−	−	Alcoholism
11	33	+	−	+	+	+	+	−	+	+	+	+	+	+	+	canabis	+	Narcissistic personality disorder
12	18	−	+	+	+	+	+	−	−	−	+	+	+	+	−	−	+	–
13	26	+	+	+	+	+	+	−	+	−	+	+	+	+	+	Amphe−tamine	+	F19; ADHS, Polytox
14	24	+	+	+	−	+	−	+	−	+	+	+	+	−	−	−	−	Pregnancy
15	52	+	−	−	+	+	+	+	+	−	−	−	+	+	+	Temesta	+	F60.30; F33.1; F10.21; Z56/Z59/Z63
16	23	+	+	+	−	+	+	−	−	+	+	+	+	+	−	−	+	–
17	24	+	+	+	−	+	+	+	−	−	+	+	+	+	−	−	+	–
18	26	−	+	+	−	+	+	+	+	−	+	+	+	+	−	−	+	–
19	45	+	+	+	−	−	+	−	+	+	−	−	−	−	−	−	−	F61
21	22	−	+	+	+	+	+	−	−	−	+	+	+	+	+	−	−	–
22	36	+	+	+	−	+	+	−	+	+	+	+	+	−	−	−	−	–
23	22	+	+	−	+	−	+	+	+	−	+	+	−	+	+	+	+	F33.1
24	49	−	−	+	+	+	+	+	+	+	−	+	+	+	−	−	−	–
25	38	−	+	+	+	+	+	−	+	+	−	+	+	+	+	−	−	–
26	20	+	−	+	−	+	+	−	+	−	+	+	−	+	−	−	+	–

^a^ ASIB: acute self injuring behavior; PSIB: prior self abusive behavior; SB: suicidal background; NC: nicotine consumption; AA: alcohol abuse; ADA: additional drug abuse; PTE: prior traumatic experiences; F10: mental and behavioral disorder due to alcoholism; F42.2: compulsive minds and—acts, mixed; F19: psychic and behavioral disorder due to frequent substance abuse and consumption of additional psychotropic substances; F60.30: emotionally instable personality disorder: impulsive type; F33.1: recurrent depressive disorder, actual moderately episode; F10.21: psychic and behavioral disorder due to abuse of psychotropic substances; dependence syndrome; Z56: problems related to employment and unemployment; Z59: problems related to housing and economic circumstances; Z63: other problems related to primary support group, including family circumstances.

### 3.2. Methylation Analysis

DNA methylation levels of two *rDNA* regions (promoter and *5′ETS*) and of *PRIMA1* were determined by bisulfite pyrosequencing [13,19,57]. Bisulfite treatment of genomic DNA was done as described previously [19]. Bisulfite treatment was carried out with the EpiTect Kit (Qiagen, Hilden, Germany) according to the manufacture’s protocol. For pyrosequencing 100 ng of bisulfite treated DNA was amplified in a reaction buffer containing 0.2 mM dNTP mix, 1.5 mM MgCl_2_ and 1.5 U Taq polymerase for 40 cycles with 10 pmol of each forward and biotinylated reverse primer and sequenced with an internal primer (Table 3). Pyrosequencing was performed in PyroMark Q24 according to the PyroMark Gold Q24 Reagents Handbook (Qiagen, Hilden, Germany). Pyrosequencing was performed in two to three independent bisulfite reactions and the average methylation frequency for each CpG site and *rDNA* region was calculated.

**Table 3:** Primers.

Primer	Sequence (5′–3′)	Region
PROL	GTTTTYGTTGTGAGTTAGGTAGAGTTT	*rDNA* promoter
PROF/PROFBIO	AAAAAAACRTCCCCAACCTCC	*rDNA* promoter
PROSEQ	GGTTTATGTGGGGGAGAGGTTGT	*rDNA* promoter
FETS	GTAGGGTTTTTTTTTTTTTTTAGGgGTTTT	*5′ETS*
LETS	CTAAAAAAAACTTTTCTCACCcAAAATAAA	*5′ETS*
PRIMABSU	GGTTGGTTTTAAATGGGGGTTGTT	*PRIMA1*
PRIMABIO3	ACCTCATTACRCACACTACAACATAAA	*PRIMA1*

### 3.3. Statistical Evaluation

Statistical analyses were performed using Excel (Microsoft, Redmond, WA, USA). All reported *p*-values are considered significant for *p* ≤ 0.05.

## 4. Conclusions

The present study is the first comparative analysis of DNA methylation of *rDNA* and *PRIMA1* in borderline personality disorder (BPD). In summary, we were able to show a significantly increased methylation of the *PRIMA1* promoter in peripheral blood samples of patients with BPD compared to controls. Interestingly, a decrease in the methylation level of the distal promoter and 5′ETS region of *rDNA* was revealed in the BPD patients. However, the small and unequal sample size and liberal *p*-value limit the interpretation of the data. Thus, it will be interesting to extend analysis to a larger set of patients and to elucidate whether significant correlations between methylation levels and clinical parameters are present. Additionally, it will be fascinating to study the factors involved in the alteration in the pathway of DNA methylation during the pathogenesis of BPD.
